# Discovery of pathway biomarkers from coupled proteomics and systems biology methods

**DOI:** 10.1186/1471-2164-11-S2-S12

**Published:** 2010-11-02

**Authors:** Fan Zhang, Jake Y Chen

**Affiliations:** 1 Indiana University School of Informatics, Indianapolis, IN 46202; 2 Dept. Computer and Information Science, Purdue School of Science, Indianapolis, IN 46202; 3 Indiana Center for Systems Biology and Personalized Medicine, Indianapolis, IN 46202

## Abstract

**Background:**

Breast cancer is worldwide the second most common type of cancer after lung cancer. Plasma proteome profiling may have a higher chance to identify protein changes between plasma samples such as normal and breast cancer tissues. Breast cancer cell lines have long been used by researches as model system for identifying protein biomarkers. A comparison of the set of proteins which change in plasma with previously published findings from proteomic analysis of human breast cancer cell lines may identify with a higher confidence a subset of candidate protein biomarker.

**Results:**

In this study, we analyzed a liquid chromatography (LC) coupled tandem mass spectrometry (MS/MS) proteomics dataset from plasma samples of 40 healthy women and 40 women diagnosed with breast cancer. Using a two-sample t-statistics and permutation procedure, we identified 254 statistically significant, differentially expressed proteins, among which 208 are over-expressed and 46 are under-expressed in breast cancer plasma. We validated this result against previously published proteomic results of human breast cancer cell lines and signaling pathways to derive 25 candidate protein biomarkers in a panel. Using the pathway analysis, we observed that the 25 “activated” plasma proteins were present in several cancer pathways, including ‘Complement and coagulation cascades’, ‘Regulation of actin cytoskeleton’, and ‘Focal adhesion’, and match well with previously reported studies. Additional gene ontology analysis of the 25 proteins also showed that cellular metabolic process and response to external stimulus (especially proteolysis and acute inflammatory response) were enriched functional annotations of the proteins identified in the breast cancer plasma samples. By cross-validation using two additional proteomics studies, we obtained 86% and 83% similarities in pathway-protein matrix between the first study and the two testing studies, which is much better than the similarity we measured with proteins.

**Conclusions:**

We presented a ‘systems biology’ method to identify, characterize, analyze and validate panel biomarkers in breast cancer proteomics data, which includes 1) t statistics and permutation process, 2) network, pathway and function annotation analysis, and 3) cross-validation of multiple studies. Our results showed that the systems biology approach is essential to the understanding molecular mechanisms of panel protein biomarkers.

## Background

Breast cancer is worldwide the second most common type of cancer after lung cancer. According to the American Cancer Society, approximately 192,370 women in the US will be diagnosed with breast cancer in 2010, and about 40,170 women will die from the disease.

Molecular biomarkers have become increasingly important clinical tools for cancer screening, diagnosis, treatment customizations. There has been an increasing number of research reports on developing breast cancer biomarkers, especially in blood [1] . Many molecular biomarkers with expression level changes have been identified in breast cancer tissue samples or blood, for example, *HER2*[2] , *PNCA*[3] , *Lipofilin B*[4] , *Cyclin D1*[5] , *CEACAM6*[6] , *Osteopontin-c*[7] , *RCP*[8] , and *FOXA1*[9] .

Most current breast cancer biomarker identification is achieved using functional genomics studies of established breast cancer cell lines [10][11][12][13][14] . Cell lines are widely used in many aspects of laboratory research and particularly as *in vitro* models in cancer research. They have a number of advantages, including being easy to access and offering “clean” results with statistically significant signals. However, human systems are quite complex [15] , and many candidate biomarkers discovered in cell lines do not readily transfer to human tissues or blood, in which clinical testing will be performed. Therefore, profiling human plasma using proteomics techniques offers an appealing alternative to cell lines or tissue biospecimens in developing protein biomarkers [16] , although the debate over this issue is heated [17] .

The question whether protein biomarker identified in blood can be valuable rests primarily on our ability to address the complexity associated with the human plasma proteome. The inherent presence of measurement noise, inconsistencies due to individual differences, and sample biases of the plasma proteomics approach have been reported [18] . However, our recent studies also showed, by collecting plasma proteomics into a common proteomics data repository, the HIP2 database [19] , we could start to reduce the perceived coverage biases for plasma proteomics, and explore a promising goldmine of candidate cancer biomarkers and drug targets [20] . In addition, bioinformatics and systems biology techniques can help reduce this complexity significantly. For example, one can use plasma proteomics to derive breast cancer candidate protein markers and then use gene expression mapping to validate candidate protein biomarkers that are known to be secreted. One can also use advanced visualization or network biology techniques such as [21][22] to model and monitor global patterns of changes in proteomics, instead of candidate biomarkers at the individual protein level [23] .

In this paper, we adopted a systems biology approach to the study of panel protein biomarker discovery in breast cancer using plasma. For polygenic diseases such as breast cancer and a complex detection platform such as human blood, we recognize that a single protein biomarker approach using “expressions” will not suffice for the high performance requirement of breast cancer screening and diagnosis. Therefore, by enlisting multiple proteins as analytes that are mechanistically linked to breast cancer pathways or functional networks, we believe that the chance of success would be higher than the simpler conventional single-marker approach.

Our computational analysis involves several steps. First, we used a t-statistics and permutation procedure to identify protein biomarker candidates that are significantly differentially detected among different individual plasma samples between the case and the control for breast cancer. Second, we performed an extensive literature curation to determine the constituents of the plasma protein biomarker panel. Third, we performed gene ontology analysis and pathway analysis to validate the list to reveal the intricate breast cancer related molecular mechanism that exists among the protein biomarkers on the panel. Fourth and last, we validated our results using two independent publicly-available breast cancer proteomics datasets derived from other groups. Our results showed that such an integrative systems approach is essential to development and validation of panel protein biomarkers that may withstand rigorous testing for the future steps.

## Results

### Normality test

**Figure 1:**
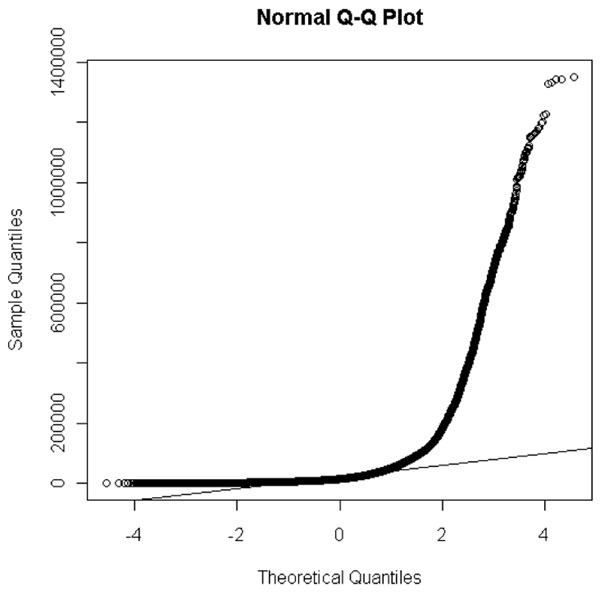
Q-Q Plot for Data from 40 Health women in Study A

The plasma proteome profiling dataset in Study A contains 4832 peptides, two states (health and breast cancer) and 40 samples each state. Q-Q plot and one sample Kolmogorov-Smirnov test in Figure 1 showed that the log2 transformation intensity values for all 4832 peptides from 40 health women are not normally distributed (One-sample Kolmogorov-Smirnov test, D = 0.0419, p-value < 2.2e-16). We also found the intensity values from 40 women diagnosed with cancer in Study A, women diagnosed with cancer from Study B and C, and healthy women form Study B and C are likewise not normally distributed.

### Pathway analysis and gene ontology categorization of significant proteins

4832 peptides in the Study A were mapped to 1422 proteins by searching against the IPI database. Using a t statistics and permutation process described in the Methods section and setting a p-value cut-off of 0.001 after initial ANOVA analysis of mass spectra data, we identified 254 statistically significant differentially expressed proteins (PFER=1.422, FDR=0.0056), among which 208 are over-expressed and 46 are under-expressed in breast cancer plasma. Compared to the results of traditional statistical testing (PFER=2.5596, FDR=0.01), our results show that the coupled statistical process outperforms the sensitivity of a parametric traditional statistical test that requires strong and sometimes untenable data assumptions since it is non-parametric and requires no assumption about the distribution under the null hypothesis.

**Figure 2:**
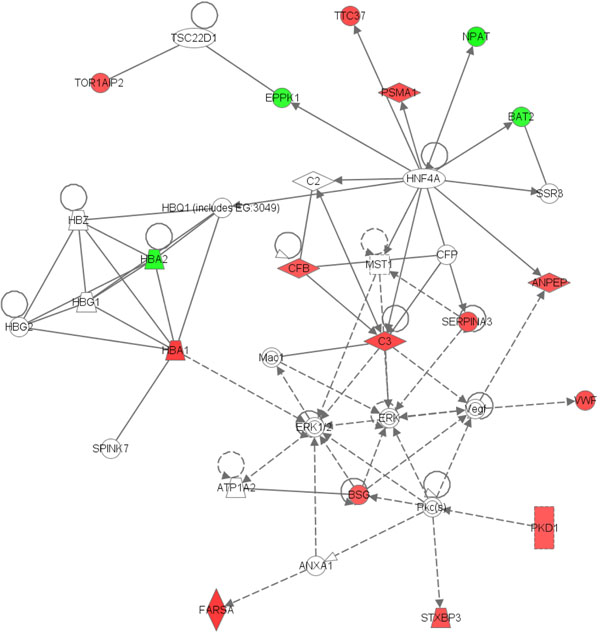
**The 25 Proteins Are Involved in an Endocrine System Disorders Network.** Red stands for over-expressed and green for under-expressed

**Figure 3:**
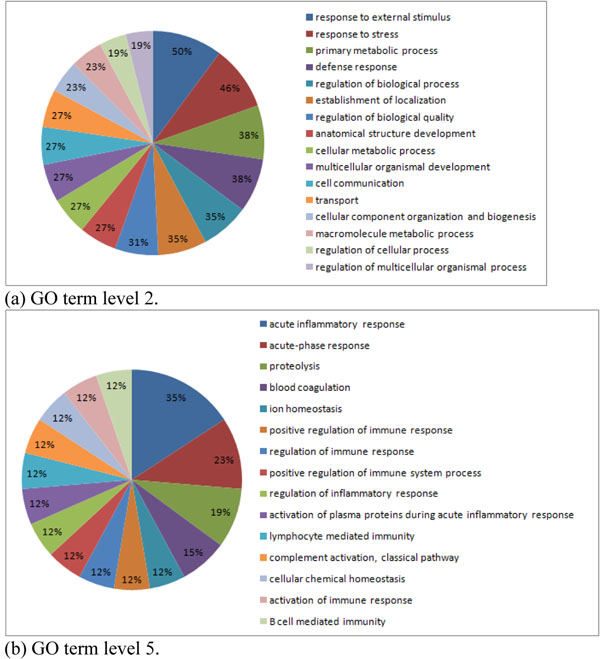
Gene Ontology Biological Processes Enrichment Analysis for 25 Protein Biomarkers.

A comparison of the set of 254 proteins with published findings from proteomic analysis of human breast cancer cell lines yielded 25 differentially expressed proteins in common. Top networks were identified by using Ingenuity Pathway Analysis (Table 1, and Figure 2). Figures 3a and 3b quantified the biological significance of gene ontology biological processes

**Table 1:** Top Networks Involved

Primary Network Functions	Computed Score	Molecules in Network
Endocrine System Disorders, Metabolic Dis-ease, Antigen Presentation	41	17

Cell-To-Cell Signaling and Interaction, Tissue Development, Hematological Disease	13	7

Gene Expression, Cancer, Dermatological Diseases and Conditions	2	1

Cardiac Arteriopathy, Cardiovascular Disease, Genetic Disorder	2	1

### Cross-validation of candidate biomarkers

**Figure 4:**
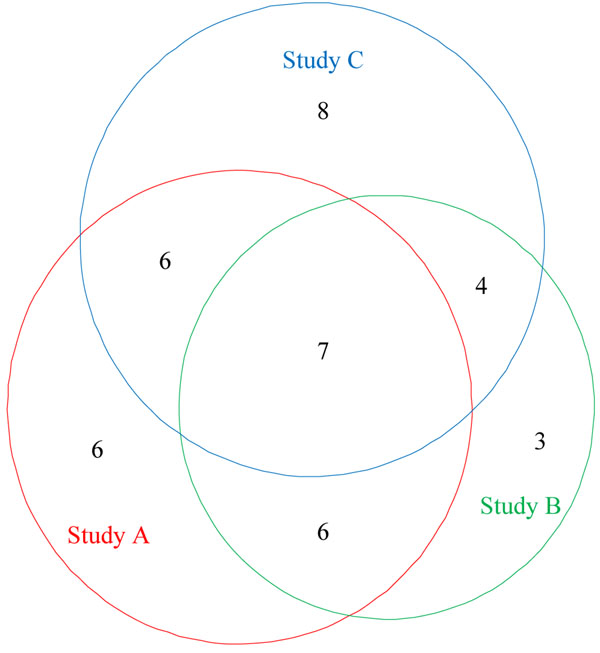
**Venn Diagram of Markers** Venn diagram indicating the degree of overlap among the markers identified from three data sets: Study A, B and C.

In order to validate our computational results, the same methods and procedures we used in Study A were applied to Studies B and C. As shown in the Venn diagram (Figure 4), 20 candidate protein biomarkers were identified in Study B, of which 13 were found in common with study A, and 25 candidate protein biomarkers were identified in Study C, of which 13 were found in common with study A. The similarity measurements with the protein method are 40% for biomarker sets from Study A and Study B and 35% for biomarker sets from Study A and Study C.

**Figure 5:**
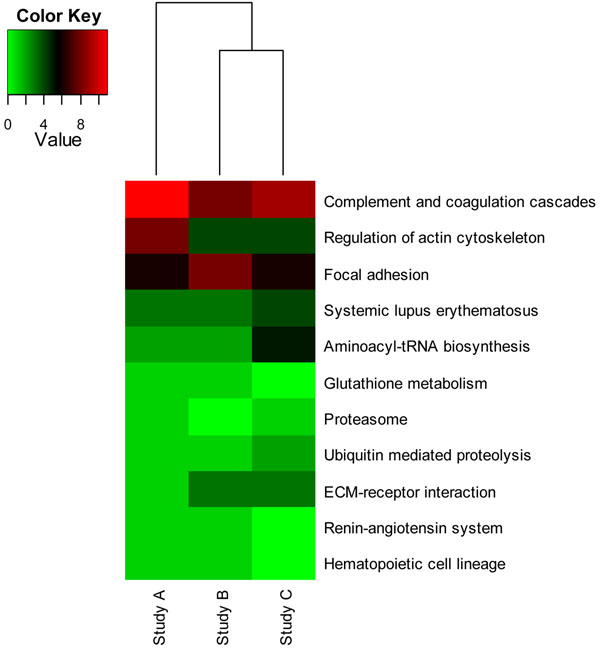
**Heatmap of Pathway-Protein Frequency Count Matrix** Vertical axis represents pathway and horizontal axis represents studies. Each cell corresponds to the count of represented proteins in pathway for the corresponding Study.

The pathway-protein association matrix is shown in the Additional file 1. The top three pathways: ‘Complement and coagulation cascades’, ‘Regulation of actin cytoskeleton’, and ‘Focal adhesion’ are ranked top in all three studies (Figure 5). The similarity measurements with the pathway-protein matrix method are 86% for biomarker sets from Study A and Study B and 83% for biomarker sets from Study A and Study C.

Using Ingenuity Pathway Analysis and DAVID GO analysis, we also found that biomarkers identified from the Study B and Study C have similar network and function as the 25 candidate protein biomarkers identified from study A.

From the cross-validation offered by our ‘systems biology’ approach, we found similar pathway, network and function in those biomarkers identified from the three studies. The systems biology approach helps to unravel the intricate pathways, networks and functional contexts in which genes or proteins function and is essential to the understanding molecular mechanisms of panel protein biomarkers.

## Discussion

### Permutation test

Most of protein identification methods were based on fold change. Fold change is easy to use and implement, fast and works with any number of arrays, but it does not take the variability of a protein into account and offers no significance measure of p-value. In this paper, we used a t-statistics to calculate the initial p-value that can takes the variance into account.

Theoretically, the t-test can be used even if the sample sizes are very small (e.g., as small as 10; some researchers claim that even smaller samples are manageable), as long as the variables are normally distributed within each group and the variation of scores in the two groups is not reliably different. If populations from which data to be analyzed by a t-test were sampled violate one or more of the t-test assumptions, the results from the analysis may be incorrect or misleading. For example, if the assumption of independence is violated, then the two-sample unpaired t-test is simply not appropriate. If the assumption of normality for the t-test is violated, or outliers are present, then the t-test may not be the most powerful test available. However, our plasma protein profiling shows no normal distribution (Figure 1). In order to detect a true difference between health and control samples, a permutation process was used, and should be reliable regardless of whether or not the sample distribution is known.

Our results showed that the permutation test was very similar to the t-test in its p-value estimate. The t-test is a parametric test and the permutation process is non-parametric. By using the permutation test we made no assumption about the distribution under the null hypothesis. Usually, the assumptions in the null hypothesis are weakened, and it becomes harder to reject. The permutation process rivals the sensitivity of a parametric t-test assuming equal variances.

To compare the power of the t-test and permutation test, that is, how likely they are to reject the null hypothesis when an alternate hypothesis is true, we assume specific distributions for the alternate hypothesis. For the t-test, the most natural alternate hypothesis is that the two samples are from different normal distributions. For large samples, it has been shown that the power of the permutation process using the difference in sample means is equal to the t-test for normally distributed alternates.

We used quantitative method to compare the results of ttperm function in the Category package of the R language using the method described in the method section. All 16 biomarkers identified using ttperm function are among the 25 panel biomarkers identified by our permutation test method in Study A. 13 of them are identified in Study C. Comparing them with the 20 candidate protein biomarkers we identified from study B, there are only 7 in common. But the remaining 9 proteins not identified by our permutation method are also not chosen as candidate protein biomarkers by previous findings using breast cell lines [10][13][14][24]. Gene ontology analysis using the 16 biomarkers showed that response to external stimulus was annotated, but cellular metabolic process was not, and acute inflammatory response was annotated but proteolysis not. The cellular metabolic process (proteolysis) annotated by our permutation method was reported to be related to cancer progression [11] . All the results show that our permutation test method is highly robust to the equality of the variances, regardless of whether the same sizes are similar and carries more conviction than the other permutation test which doesn’t consider the affect of variance inequality.

### Candidate protein biomarkers identified

A total of 416 peptides were identified from analyzing the plasma protein profiling from 40 women diagnosed with breast cancer and from 40 healthy women using the permutation test, corresponding to 254 unique UNIPROT protein names. A comparison of the 254 proteins with previously published findings from proteomic analysis of human breast cancer cell lines yielded 25 candidate protein biomarkers. The 25 proteins were categorized by their interactive pathway, network and annotated biological process on Gene Ontology.

An interesting finding from Pathway Analysis is that those top networks shown in the Table 1 and Figure 1, especially the top 1 network (Endocrine System Disorders, Metabolic Disease, Antigen Presentation) and top 3 pathways (‘Complement and coagulation cascades’, ‘Regulation of actin cytoskeleton’, and ‘Focal adhesion’), are validated by our B and C dataset results, and are similar to previously reported works [25][26][27][28] . For example, Ana-Teresa et al. studied 12 candidate genes that are implicated in the etiology of breast cancer and found these genes are functionally involved in complement and coagulation cascades pathway [29] . Carol et al. reported that the cell migration in breast cancer lines can also be regulated by actin cytoskeleton dynamics [30] . And Michael et al. reported that increased focal adhesion kinase expression correlates with TGF-β1-mediated activation of p38 MAPK in metastatic human breast cancer cells and concluded that focal adhesion is essential in mediating oncogenic signaling by transforming growth factor (TGF)-β [31] .

Another interesting finding from our Gene Ontology work is the role of cellular metabolic process and response to external stimulus (especially proteolysis and acute inflammatory response) in Figure 3a and 3b in breast cancer was also reported by other authors. For example, cancer, like other diseases, is accompanied by strong metabolic disorders [11] . And It was also reported that stress and external stimulus such as microbial infections, ultraviolet radiation, and chemical stress from heavy metals and pesticides affect the progression of breast cancer [32] .

## Conclusions

254 statistically significantly differentially expressed proteins between 40 healthy women and 40 women diagnosed with breast cancer were identified from initial LC-MS/MS experiments using a t statistics and permutation process which is useful in independent two-sample hypothesis testing. Top breast cancer activated networks and pathways were identified through systems biology approach. 25 candidate protein biomarkers were validated from the pathway/network analysis, literature curation from previous published findings in breast cell lines, and two additional studies. Gene ontology analysis confirmed that cellular metabolic process and response to external stimulus (especially proteolysis and acute inflammatory response) were enriched in the 25 protein biomarker panel. Pathway analysis identified three top enriched pathways: ‘Complement and coagulation cascades’, ‘Regulation of actin cytoskeleton’, and ‘Focal adhesion’. Our approach integrating computing, basic biomedical research, and clinical applications promises to be able to “translate” between scientific innovations and clinical diagnostic needs for breast cancer. Assay Development and Clinical Trials for Panel Biomarker from breast cancer patients are needed to assess which of the identified proteins may have diagnostic utility.

## Methods

**Figure 6:**
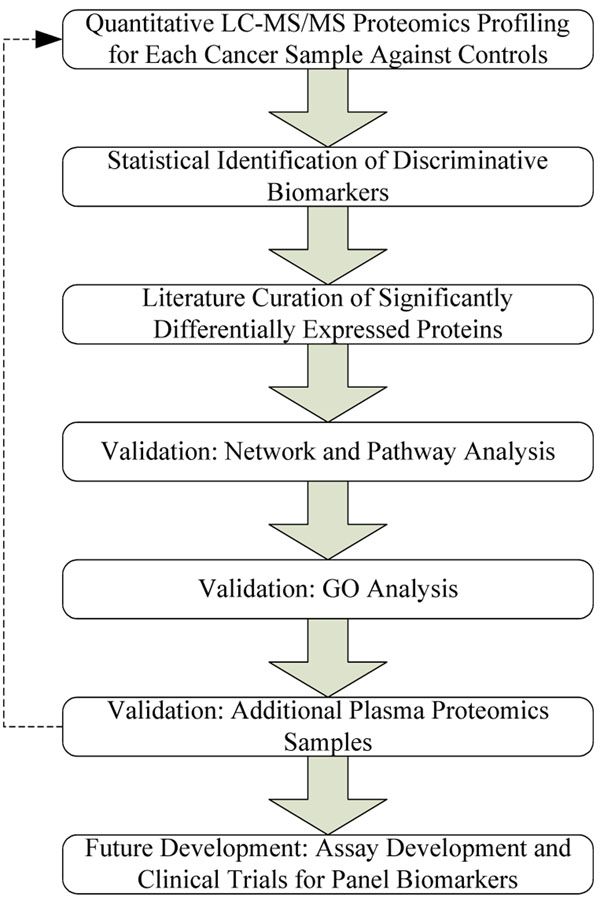
**Schema of Methods** First, proteins were prepared and subjected to LC/MS/MS analysis. Next, a permutation procedure was used to determine the p-value. Next, 4 previously published proteomic studies of breast cancer cell lines were used for comparison. Then, top networks and pathways were identified with Ingenuity Pathways analysis, KEGG and HPD pathway databases. And Level 2 and 5 of biological process in gene ontology are mainly studied. And then, two testing cancer dataset were used to validate the result. Last, assay development and clinical trials for panel biomarkers are planned for the future.

Biomarker identification and characterization holds great promise for more precise diagnoses and for tailored therapies. The heterogeneity of human cancers and unmet medical needs in these diseases provides a compelling argument to focus biomarker development in cancer. Mass spectrometry based proteomics approaches have provided insight into biomarkers of cancer and other diseases with femtomole sensitivity and high analytical precision. The schema of methods in this paper is shown in the following Figure 6.

First, proteins were prepared and subjected to LC/MS/MS analysis. Samples were run on a Surveyor HPLC (ThermoFinnigan) with a C18 microbore column (Zorbax 300SBC18, 1 mm × 5 cm). All tryptic peptides (100 μL or 20 μg) were injected onto the column in random order. Peptides were eluted with a linear gradient from 5% to 45% acetonitrile developed over 120 min at a flow rate of 50 μL/min, eluant was introduced into a ThermoFinnigan LTQ linear ion-trap mass spectrometer. The data were collected in the “triple-play” mode (MS scan, Zoom scan, and MS/MS scan). Next, a permutation procedure was used to determine the p-value. The 80 samples for each peptide were permuted 100,000 times and the complete set of t-tests between the first 40 values and the last 40 values, was performed for each permutation. Next, 4 previously published proteomic studies of breast cancer cell lines were used for comparison. Then, top networks and pathways were identified with Ingenuity Pathways analysis, KEGG and HPD pathway databases. And Level 2 and 5 of biological process in gene ontology were mainly studied. And then, two testing cancer dataset were used to validate the result. Last, assay development and clinical trials for panel biomarkers are planned for the future.

### Database

Plasma protein profiles were collected in three batches, which we refer to as Studies A, B and C. All 3 of these studies were processed in the same laboratory but at different times. Each sample was analyzed in a single batch by mass spectrometry. In both Studies A and B, 80 plasma samples were collected (40 samples collected from women with breast cancer and 40 from healthy volunteer woman who served as controls). In Study C, 40 plasma samples were collected (20 samples collected from women with breast cancer and 20 from healthy volunteer woman who served as controls). The demography and clinical distribution of breast cancer stages/subtypes for study A, B and C are comparable, although the total sample number of Study C is somewhat smaller than Study A and B.

We compared our results with 4 previously published proteomic studies of breast cancer cell lines. Their methods and results presented in peer reviewed journals [10, 12-14] have established a high reliability. A total of 3085 protein biomarkers were identified from the five breast cell lines, MCF-10A, BT474, MDA-MB-468, MD-MB-468, and T47D/MCF7 in their papers.

### Protein identification and quantification

For protein identification, Tryptic peptides were analyzed using Thermo-Finnigan linear ios-trap mass spectrometer (LTQ) coupled with a HPLC system. Peptides were eluted with a gradient from 5 to 45% Acetonitrile developed over 120 minutes and data were collected in the triple-play mode (MS Scan, zoom scan, and MS/MS scan). The acquired raw peak list data were generated by XCalibur (version 2.0) using default parameters and further analyzed by the label-free identification and quantitative algorithm using default parameters described by Higgs et al [33] . MS database searches were performed against the combined protein data set from International Protein Index (IPI; version 3.60) and the non-redundant NCBI-nr human protein database (updated 2009), which totaled 22,180 protein records. Carious data processing filters for protein identification were applied to control false-discovery rate at below 5% levels.

For protein quantification, first, all extracted ion chromatograms (XICs) were aligned by retention time. Each aligned peak were matched by precursor ion, charge state, fragment ions from MS/MS data, and retention time within a one-minute window. Then, after alignment, the area-under-the-curve (AUC) for each individually aligned peak from each sample was measured, normalized, and compared for relative abundance—all as described in [33] . Here, a linear mixed model generalized from individual ANOVA (Analysis of Variance) was used to quantify protein intensities. In principle, the linear mixed model considers three types of effects when deriving protein intensities based on weighted average of quantile-normalized peptide intensities: 1) group effect, which refers to the fixed non-random effects caused by the experimental conditions or treatments that are being compared; 2) sample effect, which refers to the random effects (including those arising from sample preparations) from individual biological samples within a group; 3) replicate effect, which refers to the random effects from replicate injections from the same sample preparation.

### “Systems biology” analysis

We applied a “systems biology” approach to the study of panel biomarker discovery problem in breast cancer proteomics data study in this study. Our strategies for analyzing potentially noisy proteomics data set are three-fold. First, we used a t-statistics and permutation procedures to calculate p-value for proteins changed in all samples, instead of fold change or t-test for a given sample that were commonly used in previous studies. This allowed us to better filter the proteomics results. Second, we used extensive literature curation to focus only on breast cancer relevant differentially expressed proteins. This literature curation step allowed us to concentrate on breast cancer relevant signals, with generally noisy proteomics data sets. Third, we used gene ontology analysis and pathway analysis to identify and validate correlated changes due to cancer cell signaling that may, individually, elude the detection.

### T statistics and permutation process

Our test statistic for study A and B is a mean of 40 values (protein intensities in health samples) minus the mean of another 40 values (protein intensities in cancer samples). For study C, the total sample number is 40 with 20 healthy samples and 20 cancer samples. A permutation procedure was used to determine the p-value for each protein, representing the chance of observing a test statistic at least as large as the value actually obtained. The 80 samples for each protein in Study A and B (40 samples for Study C) were permuted 100000 times and the complete set of t-tests was performed for each permutation. The permutation p-value for a particular protein is the proportion of the permutations in which the permuted test statistic exceeds the observed test statistic in absolute values. We chose a significance level α=0.001 to select proteins where we estimated significant differences in the health and cancer sampled. The corresponding “per-family Type 1 error rate, PFER”, that is, the expected number of false positives for such a multiple test procedure is PFER = number of genes x 0.001. Alternatively, the nominal “false discovery rate, FDR”, or expected proportion of false positive among the genes declared differentially expressed, is FDR = PFER/number of genes declared expressed.

### Network and function annotation analysis

Ingenuity Pathway Analysis was used for building network. DAVID database was used to study level 2 and 5 of biological process in gene ontology. Fisher’s exact test is used to test the statistical significance for association between the gene list with expression changes and the function set [34] .

### Pathway-protein association matrix

Pathway comparison are performed using the following databases: Kyoto Encyclopedia of Genes and Genomes (http://www.genome.ad.jp/kegg/) [35] and HPD [36] . The visualization for the pathway-protein association matrix was created by Excel 2007 VBA.

### Pathway-protein frequency count matrix

A pathway-protein frequency count matrix (PPFCM) contains pathway on vertical axis and studies represented on horizontal axis. Each cell of PPFCM corresponds to the count of represented proteins in pathway for the corresponding Study.

### Biomarker set similarity

We presented two approaches to measure the similarity of biomarker sets: 1) protein method and 2) pathway-protein matrix method.

The biomarker set similarity measure with the protein method can be defined as the extent of overlaps, e.g., common number of biomarkers, shared between two different biomarker sets. The set-set similarity score *S_i,j_* is defined as

     (1)

where, *N* denotes total number of biomarker sets. *P_i_* and *P_j_* denote two different biomarker sets, while *|P_i_|* and *|P_j_|* are the numbers of biomarkers in these two sets. Their intersection *P_i_* ∩ *P_j_* is the set of all biomarkers that appear in both *P_i_* and *P_j_*, while their union *P_i_* ∪ *P_j_* is a set of all biomarkers either appearing in the *P_i_* or in the *P_j_.* Duplicates are eliminated in the intersection set and union set.

The biomarker set similarity measure with the pathway-protein matrix method can be defined as the correction coefficient of the number of represented biomarkers in pathways for sets. The higher the correction coefficient is, the more similar the two sets. The equation is expressed as

     (2)

where *Corr* is the Pearson correlation coefficient, *Q_i_* is the biomarker numbers in pathways for set *i*, *N* denotes total number of biomarker sets.

## Competing interests

The authors declare that they have no competing interests.

## Authors' contributions

JYC presented the idea and constructed the general design. FZ collected data, performed the statistical analyses and wrote the paper. All authors read and approved the final manuscript.

## Supplementary Material

Additional file 1 Pathway-Protein Association Matrix Click here for file

## References

[B1] Brooks M Breast cancer screening and biomarkers Methods Mol Biol 2009 472 307 321 19107439 10.1007/978-1-60327-492-0_13

[B2] Carlsson J Nordgren H Sjostrom J Wester K Villman K Bengtsson NO Ostenstad B Lundqvist H Blomqvist C HER2 expression in breast cancer primary tumours and corresponding metastases. Original data and literature review British journal of cancer 2004 90 12 2344 2348 15150568 10.1038/sj.bjc.6601881 PMC2409528

[B3] Malkas LH Herbert BS Abdel-Aziz W Dobrolecki LE Liu Y Agarwal B Hoelz D Badve S Schnaper L Arnold RJ A cancer-associated PCNA expressed in breast cancer has implications as a potential biomarker Proceedings of the National Academy of Sciences of the United States of America 2006 103 51 19472 19477 17159154 10.1073/pnas.0604614103 PMC1697829

[B4] Culleton J O’Brien N Ryan BM Hill AD McDermott E O’Higgins N Duffy MJ Lipophilin B: A gene preferentially expressed in breast tissue and upregulated in breast cancer International journal of cancer 2007 120 5 1087 1092 10.1002/ijc.22471 17163411

[B5] Colombo M Giarola M Mariani L Ripamonti CB De Benedetti V Sardella M Losa M Manoukian S Peissel B Pierotti MA Cyclin D1 expression analysis in familial breast cancers may discriminate BRCAX from BRCA2-linked cases Mod Pathol 2008 21 10 1262 1270 18327210 10.1038/modpathol.2008.43

[B6] Lewis-Wambi JS Cunliffe HE Kim HR Willis AL Jordan VC Overexpression of CEACAM6 promotes migration and invasion of oestrogen-deprived breast cancer cells Eur J Cancer 2008 44 12 1770 1779 18614350 10.1016/j.ejca.2008.05.016 PMC2778047

[B7] Mirza M Shaughnessy E Hurley JK Vanpatten KA Pestano GA He B Weber GF Osteopontin-c is a selective marker of breast cancer International journal of cancer 2008 122 4 889 897 10.1002/ijc.23204 17960616

[B8] Rao PN Levine E Myers MO Prakash V Watson J Stolier A Kopicko JJ Kissinger P Raj SG Raj MH Elevation of serum riboflavin carrier protein in breast cancer Cancer Epidemiol Biomarkers Prev 1999 8 11 985 990 10566553

[B9] Thorat MA Marchio C Morimiya A Savage K Nakshatri H Reis-Filho JS Badve S Forkhead box A1 expression in breast cancer is associated with luminal subtype and good prognosis J Clin Pathol 2008 61 3 327 332 18037662 10.1136/jcp.2007.052431

[B10] Adam PJ Boyd R Tyson KL Fletcher GC Stamps A Hudson L Poyser HR Redpath N Griffiths M Steers G Comprehensive Proteomic Analysis of Breast Cancer Cell Membranes Reveals Unique Proteins with Potential Roles in Clinical Cancer J Biol Chem 2003 278 8 6482 6489 12477722 10.1074/jbc.M210184200

[B11] Bullinger D Neubauer H Fehm T Laufer S Gleiter CH Kammerer B Metabolic signature of breast cancer cell line MCF-7: profiling of modified nucleosides via LC-IT MS couplin BMC Biochem 2007 8 25 18047657 10.1186/1471-2091-8-25 PMC2219991

[B12] Kulasingam V Diamandis EP Proteomics Analysis of Conditioned Media from Three Breast Cancer Cell Lines: A Mine for Biomarkers and Therapeutic Targets Mol Cell Proteomics 2007 6 11 1997 2011 17656355 10.1074/mcp.M600465-MCP200

[B13] Mbeunkui F Metge BJ Shevde LA Pannell LK Identification of Differentially Secreted Biomarkers Using LC-MS/MS in Isogenic Cell Lines Representing a Progression of Breast Cancer J Proteome Res 2007 6 8 2993 3002 17608509 10.1021/pr060629m PMC2584611

[B14] Xiang R Shi Y Dillon DA Negin B Horvath C Wilkins JA 2D LC/MS Analysis of Membrane Proteins from Breast Cancer Cell Lines MCF7 and BT474 J Proteome Res 2004 3 6 1278 1283 15595738 10.1021/pr049852e

[B15] Naylor S Chen JY Unraveling human complexity and disease with systems biology and personalized medicine Personalized Medicine 2010 7 3 20577569 10.2217/pme.10.16 PMC2888109

[B16] Burdall S Hanby A Lansdown M Speirs V Breast cancer cell lines: friend or foe? Breast Cancer Res 2003 5 2 89 95 12631387 10.1186/bcr577 PMC154155

[B17] Simpson RJ Bernhard OK Greening DW Moritz RL Proteomics-driven cancer biomarker discovery: looking to the future Curr Opin Chem Biol 2008 12 1 72 77 18295612 10.1016/j.cbpa.2008.02.010

[B18] Johann DJ Jr. McGuigan MD Patel AR Tomov S Ross S Conrads TP Veenstra TD Fishman DA Whiteley GR Petricoin EF 3rd Clinical proteomics and biomarker discovery Ann N Y Acad Sci 2004 1022 295 305 15251975 10.1196/annals.1318.045

[B19] Saha S Harrison SH Shen C Tang H Radivojac P Arnold RJ Zhang X Chen JY HIP2: An online database of human plasma proteins from healthy individuals BMC Med Genomics 2008 1 12 18439290 10.1186/1755-8794-1-12 PMC2396660

[B20] Saha S Harrison SH Chen JY Dissecting the human plasma proteome and inflammatory response biomarkers Proteomics 2009 9 2 470 484 19105179 10.1002/pmic.200800507 PMC3402908

[B21] Huan T Sivachenko A Harrison S Chen JY ProteoLens: a Visual Analytic Tool for Multi-scale Database-driven Biological Network Data Mining BMC Bioinformatics 2008 9 S5 18793469 10.1186/1471-2105-9-S9-S5 PMC2537576

[B22] Chen JY Shen C Sivachenko A Mining Alzheimer Disease Relevant Proteins from Integrated Protein Interactome Data Pacific Symposium on Biocomputing ‘06 2006 11 367 378 Maui, HI 17094253

[B23] Chen JY Yan Z Shen C Fitzpatrick DP Wang M A systems biology approach to the study of cisplatin drug resistance in ovarian cancers Journal of bioinformatics and computational biology 2007 5 2a 383 405 10.1142/s0219720007002606 17589967

[B24] Alexander H Stegner AL Wagner-Mann C Du Bois GC Alexander S Sauter ER Proteomic Analysis to Identify Breast Cancer Biomarkers in Nipple Aspirate Fluid Clin Cancer Res 2004 10 22 7500 7510 15569980 10.1158/1078-0432.CCR-04-1002

[B25] Berishaj M Gao SP Ahmed S Leslie K Al-Ahmadie H Gerald WL Bornmann W Bromberg JF Stat3 is tyrosine-phosphorylated through the interleukin-6/glycoprotein 130/Janus kinase pathway in breast cancer Breast Cancer Res 2007 9 3 R32 17531096 10.1186/bcr1680 PMC1929096

[B26] Hu H Lee HJ Jiang C Zhang J Wang L Zhao Y Xiang Q Lee EO Kim SH Lu J Penta-1,2,3,4,6-O-galloyl-beta-D-glucose induces p53 and inhibits STAT3 in prostate cancer cells in vitro and suppresses prostate xenograft tumor growth in vivo Mol Cancer Ther 2008 7 9 2681 2691 18790750 10.1158/1535-7163.MCT-08-0456

[B27] Song H Jin X Lin J Stat3 upregulates MEK5 expression in human breast cancer cells Oncogene 2004 23 50 8301 8309 15378007 10.1038/sj.onc.1208026

[B28] Gemmill JA Stratton P Cleary SD Ballweg ML Sinaii N Cancers, infections, and endocrine diseases in women with endometriosis Fertil Steril 2009 19945097 10.1016/j.fertnstert.2009.07.1698 PMC2946463

[B29] Maia AT Spiteri I Lee AJ O’Reilly M Jones L Caldas C Ponder BA Extent of differential allelic expression of candidate breast cancer genes is similar in blood and breast Breast Cancer Res 2009 11 6 R88 20003265 10.1186/bcr2458 PMC2815552

[B30] Sawyer C Sturge J Bennett DC O’Hare MJ Allen WE Bain J Jones GE Vanhaesebroeck B Regulation of breast cancer cell chemotaxis by the phosphoinositide 3-kinase p110delta Cancer Res 2003 63 7 1667 1675 12670921

[B31] Wendt MK Schiemann WP Therapeutic targeting of the focal adhesion complex prevents oncogenic TGF-beta signaling and metastasis Breast Cancer Res 2009 11 5 R68 19740433 10.1186/bcr2360 PMC2790843

[B32] Nielsen NR Gronbaek M Stress and breast cancer: a systematic update on the current knowledge Nat Clin Pract Oncol 2006 3 11 612 620 17080179 10.1038/ncponc0652

[B33] Higgs RE Knierman MD Gelfanova V Butler JP Hale JE Comprehensive label-free method for the relative quantification of proteins from biological samples Journal of proteome research 2005 4 4 1442 1450 16083298 10.1021/pr050109b

[B34] Mehta CR Patel NR Tsiatis AA Exact significance testing to establish treatment equivalence with ordered categorical data Biometrics 1984 40 3 819 825 6518249

[B35] Kanehisa M Araki M Goto S Hattori M Hirakawa M Itoh M Katayama T Kawashima S Okuda S Tokimatsu T KEGG for linking genomes to life and the environment Nucleic Acids Res 2008 36 Database issue D480 484 18077471 10.1093/nar/gkm882 PMC2238879

[B36] Chowbina SR Wu X Zhang F Li PM Pandey R Kasamsetty HN Chen JY HPD: an online integrated human pathway database enabling systems biology studies BMC Bioinformatics 2009 10 Suppl 11 S5 19811689 10.1186/1471-2105-10-S11-S5 PMC3226194

